# Supplementary material for: Data Valuation with Gradient Similarity
Source: ArXiv. 2024 May 13:arXiv:2405.08217v1. Preprint. [Version 1] (PMC11118599)
Supplement: Supplement 1 [file NIHPP2405.08217v1-supplement-1.pdf]

5 Supplementary

5.1 DVGS Robustness to Hyperparameters

To test the robustness of the DVGS method with respect to algorithm hyperparameters, we performed a grid search on the ADULT dataset with 20% corrupted endogenous labels. We record the ability of DVGS to identify the corrupted labels across all tested hyperparameters. Figure 6 shows the cumulative distribution function (CDF) of the resulting AUROC values across all hyperparameters tested. Note that the AUROC metric characterizes the ability of data values to classify corrupted labels. We find that almost 85% of the tested hyperparameter configurations resulted in performances within 25% of the maximum performance, and more that 50% of the tested hyperparameters resulted in performance within 10% of the maximum performance, indicating that the DVGS method is robust to choice of hyperparameters. The hyperparameter grid search configurations are shown in Table 4.

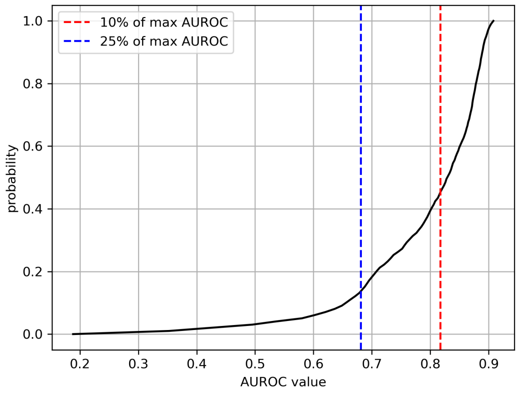

Figure 6: The cumulative distribution function (CDF) of $AUROC(c_i, -\nu_i)$ across all tested hyperparameters, where ν_i are data values generated by DVGS and c_i are the corrupted labels label. The red dashed line demarcates all AUROC values larger than this are within 10% of the max AUROC value (e.g., roughly 55% of all tested hyperparameters resulted in an AUROC value within 10% of the max AUROC).

5.2 Average Pearson Correlation (APC) metric

We compute the previously proposed Average Pearson Correlation (APC) [17] of LINCS level 4 replicates using the procedure:

For a given level 5 LINCS sample:

- Identify the level 4 bio-replicate *sample id*s that were used to generate the level 5 aggregate sample.
- Load the level 4 sample ID expression profile into memory
- Filter to select only landmark genes (978)
- Compute the average pairwise Pearson correlation of level 4 bio-replicates

As shown in Figure 7, the resulting APC distribution is skewed right, with the majority of samples having an APC less than 0.5, suggesting that most of the replicates are highly discordant. Notably, future work may wish to perform data valuation directly on the level 4 samples, which may enable researchers to "rescue" high-quality replicates, even if the replicates are highly discordant.

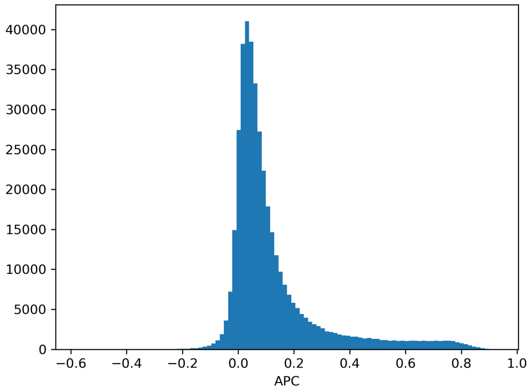

Figure 7: The Average Pearson Correlation (APC) distribution of level 5 LINCS samples.

Hyperparameter	Values	Optimal value
balanced class weights	True, False	False
dropout	0, 0.25, 0.5, 0.75	0.25
target batch size	100, 200, 400	200
similarity	Euclidean, Cosine Similarity, Dot Product, Scalar Projection	Euclidean
learning rate	1e-2, 1e-3, 1e-4	1e-3
Instance normalization	True, False	True
number of layers	1,2	1
activation function	Mish, ReLU	Mish

Table 4: The DVGS hyperparameter configurations tested in a grid search with 2 replicates per configuration.

5.3 Additional Runtime Experiments

In Figure 8 we show the experimental results of DVGS as the number of source samples increases. As expected, DVGS scales linearly with the number of source samples, divided by the period of gradient computations (T). In Figure 8b we show the ability of DVGS to classify corrupted labels, when we increase the value of T, as one would expect, the AUROC value decreases with larger T, however, the marginal decrease in performance may be worthwhile for the improvements in runtime, especially on large datasets. When applying our method to the LINCS dataset, we were able to run 500 epochs of DVGS on 710,216 source samples using a multilayer autoencoder neural network (Number parameters > 650k) in roughly 8 hours on a Nvidia 3090 GPU.

The memory requirement of the DVGS method is in many ways comparable to classical SGD optimization problems; however, the computation of high-dimensional sample-wise gradients can increase the memory requirements. Therefore, as the number of model parameters increases, the memory footprint of the sample gradients will also increase. To mitigate this issue, we chose to compute sample gradients in mini-batches, which can be manually specified to fit a given task. Reducing the source batch size will therefore reduce the memory footprint, but lead to a small increase in computation time. Additionally, the user can also choose to select a subset of all the model parameters to use for gradient computation, which will reduce memory overhead.

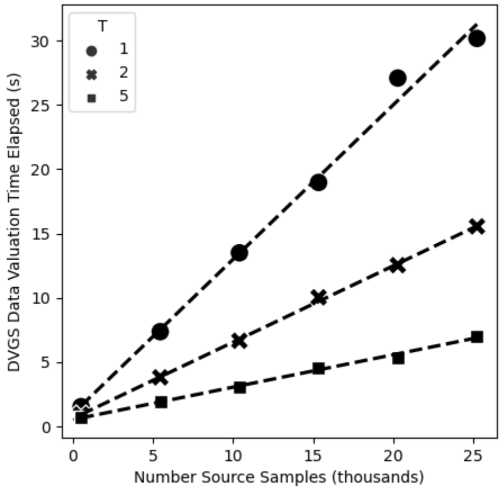

(a) DVGS runtime on the ADULT dataset when computing gradient similarities every T steps.

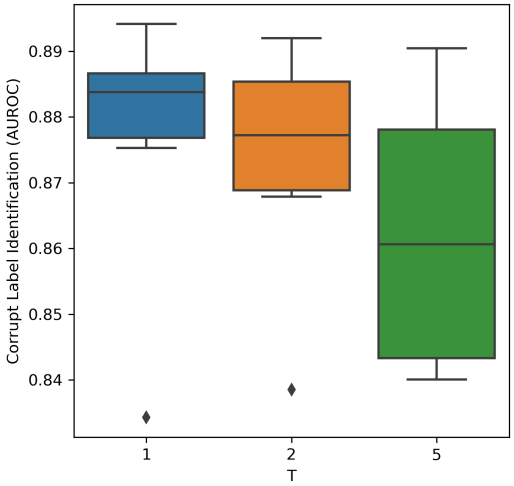

(b) Ability of DVGS to identify corrupted labels, with different values of T (period of source gradient computations).

Figure 8: The scalability and performance of the DVGS method dependant on number of source samples and the period of source similarity computations (T).
